# Brain-Inspired Affective Empathy Computational Model and Its Application on Altruistic Rescue Task

**DOI:** 10.3389/fncom.2022.784967

**Published:** 2022-07-18

**Authors:** Hui Feng, Yi Zeng, Enmeng Lu

**Affiliations:** ^1^Research Center for Brain-Inspired Intelligence, Institute of Automation, Chinese Academy of Sciences, Beijing, China; ^2^School of Artificial Intelligence, University of Chinese Academy of Sciences, Beijing, China; ^3^Center for Excellence in Brain Science and Intelligence Technology, Chinese Academy of Sciences, Shanghai, China; ^4^National Laboratory of Pattern Recognition, Institute of Automation, Chinese Academy of Sciences, Beijing, China

**Keywords:** affective empathy, mirror neuron system, spiking neural network, Artificial Pain, altruistic behavior, self-awareness

## Abstract

Affective empathy is an indispensable ability for humans and other species' harmonious social lives, motivating altruistic behavior, such as consolation and aid-giving. How to build an affective empathy computational model has attracted extensive attention in recent years. Most affective empathy models focus on the recognition and simulation of facial expressions or emotional speech of humans, namely Affective Computing. However, these studies lack the guidance of neural mechanisms of affective empathy. From a neuroscience perspective, affective empathy is formed gradually during the individual development process: experiencing own emotion—forming the corresponding Mirror Neuron System (MNS)—understanding the emotions of others through the mirror mechanism. Inspired by this neural mechanism, we constructed a brain-inspired affective empathy computational model, this model contains two submodels: (1) We designed an Artificial Pain Model inspired by the Free Energy Principle (FEP) to the simulate pain generation process in living organisms. (2) We build an affective empathy spiking neural network (AE-SNN) that simulates the mirror mechanism of MNS and has self-other differentiation ability. We apply the brain-inspired affective empathy computational model to the pain empathy and altruistic rescue task to achieve the rescue of companions by intelligent agents. To the best of our knowledge, our study is the first one to reproduce the emergence process of mirror neurons and anti-mirror neurons in the SNN field. Compared with traditional affective empathy computational models, our model is more biologically plausible, and it provides a new perspective for achieving artificial affective empathy, which has special potential for the social robots field in the future.

## 1. Introduction

Empathy is the ability to understand the state of others through observation, imagination, and inference, which is the motivation for altruistic behaviors such as consolation and aid-giving (Waal and Preston, 2017). Empathy plays a vital role in human society as it is the biological basis of social morality (Lou, 2011). In addition, empathy is cross-species (Malin et al., 2011), which maintains communication not only within the same species but also between different species. In general, empathy is fundamental to the harmonious coexistence of social groups.

Empathy is divided into cognitive empathy and affective empathy (Asada, 2015). Cognitive empathy refers to the ability that observers to imagine and infer the target's feeling or state. Affective empathy refers to the ability that observers are directly affected by the emotional state of another by matching it (Waal and Preston, 2017). Affective empathy is more fundamental and more common, and it appears in the early stage of life development and also exists in most non-human species such as rodents and birds (Shamay-Tsoory et al., 2009). Affective empathy also has essential research value in the field of social robotics as it is conducive to the communication and cooperation between robots and between humans and robots. Our work mainly focuses on the affective empathy computational model and its applications.

Emotion is an embodied internal state of the organism's brain that is formed through interaction with the environment (Lerner et al., 2015). Emotion not only represents the organism's state but also generates emotional overt actions such as facial expressions to communicate with peers and achieve affective empathy. From the psychology perspective, the observer can use the Perception-Action Mechanism (PAM) to empathize with others (Preston and de Waal, 2017). Perceiving other people's emotions will activate the same emotion representation in the observer's brain, which is equivalent to the observer experiencing that emotion. Recently, much neuroscience literature has shown that the Mirror Neuron System (MNS) supports the PAM (Rizzolatti and Sinigaglia, 2016). The MNS consists of a group of neurons with sensory-motor properties, and they are activated both during action execution and action observation (Erhan et al., 2006; Khalil et al., 2018b). The MNS a collection of Motor brain regions in the parietal and frontal lobes. In the process of affective empathy, when we perceive others' emotions, such as seeing their facial expressions or hearing them cry, we first achieve motor-level understanding through the MNS and then further achieve emotion-level understanding (Carr et al., 2003). It is worth noting that the activation patterns of the brain during experiencing own emotion and empathizing with others are different because of the presence of anti-mirror neurons (Christian and Valeria, 2010). Thus, the brain can distinguish who is the producer of emotion, which is called primary self-awareness (Lamm et al., 2011).

We argue that computational modeling of affective empathy should follow its neural mechanisms: First, the model should have an internal state similar to human emotion. Second, the model should develop its own mirror mechanism similar to the human MNS function, so as to understand others' emotions with its own experience. Third, the model should have the ability to distinguish self from others and adopt adaptive responding behaviors.

Most of the existing affective empathy computational models mainly belong to the Affective Computing field, which uses machine learning algorithms to recognize and simulate human emotion overt signals, such as facial expressions, speeches, and body movements (Claret et al., 2017; Soujanya et al., 2017; Huang et al., 2020; Lee and Kang, 2020). (Leite et al., 2014) designed an iCat robot to play chess with children. It can analyze the state of the chess game and the child's facial expressions to make a corresponding response such as encouragement. Zheng et al. (2019) proposed a children's companion robot called BabeBay, which has affective computing ability for multi-modal information such as expression, body gesture, and text, to maintain companionship with different children. These traditional approaches are only computational processing of the emotion overt signals, but the emotion overt signals are not equal to the emotion. Emotion is a complex embodied internal state. It is obvious that these approaches do not define this internal state; they treat emotion as a mere label. Therefore, we believe that these approaches are far from enough to achieve real artificial affective empathy. In addition to that, these approaches also do not follow the guidance of any neural mechanism of affective empathy. Woo et al. (2017) implemented the affective empathy process using the spiking neural network, However, this study only used brain-like modeling tools and did not involve a mirror mechanism. Watanabe et al. (2007) modeled a communication model for the virtual robot similar to the 'intuitive parenting' process between babies and their caregivers. The robot can establish the relationship between the emotions and the caregiver's facial expressions by mirror mechanism, then they can respond to the other's emotion by observing human facial expressions. But again, this study did not define emotion as an internal state of the robot, and this model cannot distinguish the producer of emotion between self and others.

In this article, we construct a more sophisticated brain-inspired affective empathy computational model. First, we constructed a computational model of emotion as an internal state: the neural mechanisms by which emotions emerge have not been well studied. The Free Energy Principle (FEP) has been proposed as a unified Bayesian interpretation of perception, learning, and action, describing the relationship between the internal model of the brain and the relevant sensations from the environment (Joffily and Coricelli, 2013). Coincidentally, emotion is a neural activity formed through the interaction of brain expectations with the real situation of the environment (Brown and Brune, 2012). Therefore, in this article, we use FEP as a theoretical basis to model a specific emotion–pain. Second, we modeled the mirror mechanism of empathy: we used a spiking neural network to reproduce the emergence process of mirror neurons and anti-mirror neurons, the process of empathizing with others through mirror mechanism, and the ability of self-other differentiation.

The contributions of our study can be summarized as follows: (1) Inspired by FEP, we modeled the Artificial Pain as an internal state. (2) We constructed an affective empathy spiking neural network (AE-SNN), which can simulate the mirror mechanism of MNS to empathize with others and have the ability of self-others differentiation. (3) We also explored the intrinsic motivations of altruistic behavior, together with the above parts, to complete the pain empathy and altruistic rescue task in the grid world.

## 2. Materials and Methods

### 2.1. The Neural Mechanism

**Figure 1:**
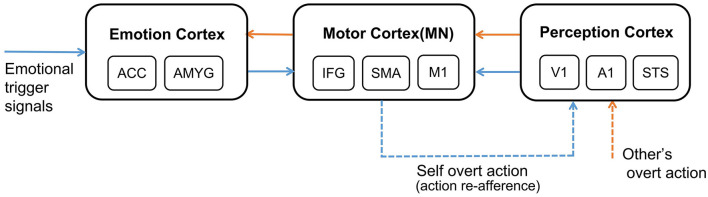
The neural mechanism of affective empathy. The brain areas contain: Emotion Cortex, Motor Cortex, and Perception Cortex. All the blue arrows represent the process of experiencing own emotions, and all the orange arrows represent the process of empathizing with others. The blue dashed arrow represents the process of action execution and action re-afference, and the orange dashed arrow represents perceiving other's overt action. There are mirror neurons (MN) in the Motor Cortex.

The whole process of affective empathy is shown in Figure 1. It involves the following brain areas: On the far left is the Emotion Cortex. Different emotional trigger signals can result in different firing patterns in this cortex, producing different emotional states. The Emotion Cortex contains many sub-regions, such as the Anterior Cingulate Cortex (ACC) and Amygdala (AMYG), which are essential components of the Limbic System (Reep et al., 2007). The ACC is often thought to be associated with pain (Corradi-Dell'Acqua et al., 2016). The AMYG is often thought to be associated with fear (Davis, 1992). In the middle is the Motor Cortex. In the affective empathy process, the Motor Cortex is mainly responsible for producing emotional overt actions (Mukamel et al., 2010), such as painful facial expressions, shouting, and crying. The Motor Cortex contains many sub-regions. Our study considers only three representative sub-regions: Inferior Frontal Gyrus (IFG), Supplementary Motor Cortex (SMA), and Primary Motor Cortex (M1). IFG is responsible for encoding the intention of actions (Jabbi, 2008). SMA is responsible for the initialization of the action sequence (Rizzolatti and Luppino, 2001). M1 guides the muscles to perform specific actions (Gazzola and Keysers, 2008). There are mirror neurons (MN) in the Motor Cortex (Erhan et al., 2013), as shown in Figure 1. On the far right is the Perception Cortex, which is used to perceive the emotional overt actions of oneself or others, such as seeing the painful facial expressions and hearing the cry. We also listed three of the most representative sub-regions in the Perception Cortex: Primary Auditory Cortex (A1) and Primary Visual Cortex (V1) perform primary processing of visual and auditory information (Zipser et al., 1996; Morosan et al., 2001). The Superior Temporal Sulcus (STS) is a high-order perception area that integrates visual and auditory information about the body and facial actions (Keysers and Gazzola, 2014). The connection between the Motor Cortex and the Emotion Cortex is bidirectional (Jabbi, 2008). The connection between the Perception Cortex and the Motor Cortex is also bidirectional (Kilner and Frith, 2007), but for the affective empathy process in our study, we only considered the connection from the Perception Cortex to the Motor Cortex.

Different emotional trigger signals will lead to the production of corresponding emotions. In this article, we explore a particular emotion–pain. External noxious stimuli specifically activate the Nociceptors in the skin of organisms, then the noxious information will be transmitted to the cerebral cortex through the Anterolateral System (Asada, 2019). In the cerebral cortex, the pain will be evoked from two aspects: the sensory discrimination component will be formed in the Somatosensory Cortex (S1, S2), and the affective and motivational component will be formed in the Anterior Cingulate Cortex (ACC) (Donald, 2000). However, this pathway is specific and formed in thousands of years of evolution (Walters and Williams, 2019). We argue that the Artificial Pain Model should not follow the physiological mechanism completely, because many intelligent agents, such as robots, do not have a physiological structure similar to organisms. We should explore the essence and significance of pain for the survival of organisms.

Broom et al. proposed that the emergence of pain was first related to physical injury in the evolutionary process (Broom, 2001). Pain is a kind of neural activity that occurs with physical injury and is preserved and internalized in the brain because of its survival benefits. In further learning, the experience of pain can also be associated with injury-related cues, such as scenes or voices of injury. When a similar cue occurs again, the brain will produce the same pain experience and avoid potential injury (Wiech and Tracey, 2013).

Certain emotions will trigger the Motor Cortex to perform corresponding emotional overt action, as shown by the blue dashed arrow in Figure 1. After experiencing own emotions, the observer can use the mirror mechanism of the Mirror Neuron System (MNS) to empathize with others. Mirror neurons are widely present in the Motor Cortex, and both the previously mentioned IFG and SMA sub-regions contain mirror neurons (Rizzolatti and Sinigaglia, 2016). MNS is formed gradually during individual development. Keysers et al. proposed that the emergence of mirror neurons is due to synchronous action execution and action re-afference. You can see or hear your emotional overt action when you execute it. This kind of perceptual input resulting from your action is called action re-afference (Keysers and Gazzola, 2014), as shown by the blue dashed arrow in Figure 1. The existence of the time overlap between action execution and action re-afference strengthens the synapse weights between the neurons in the Motor Cortex and the Perception Cortex representing the same action, and weakens the synapse weights representing different actions, resulting in some neurons of the Motor Cortex having mirror properties, namely mirror neurons. In the process of affective empathy, perceiving the emotional overt action of others will first activate the corresponding neurons in the Perception Cortex, as shown by the orange dashed arrow in Figure 1. Subsequently, the mirror neurons of the Motor Cortex that perform the same emotional overt action will fire, forming the internal motor level representation of other's emotion. The Motor Cortex is connected to the Emotion Cortex, which further activates the corresponding emotional neurons, forming an internal emotion-level representation of others' emotions, as shown by the orange solid arrow in Figure 1. For example, when a baby is experiencing emotions, it will instinctively activate the Motor Cortex to produce emotional overt actions (Yamada, 1993), such as crying or facial expressions. Babies can also perceive these emotional overt actions by their Auditory Cortex or Visual Cortex. When a baby performs a happy facial expression, their caregiver will imitate their expression so that they will also see the caregiver's expression, called Intuitive Parenting (Watanabe et al., 2007). Due to the similar activation time, the synaptic weights from the neurons in the Perception Cortex to the neurons in the Motor Cortex that represent the same emotional overt action will be strengthened, forming mirror neurons. When seeing others' facial expressions or hearing others' cries, they can understand others' emotions through the mirror mechanism.

In addition, the affective empathy network of the brain can distinguish the self from others. The reason is that the anti-mirror neurons in Motor Cortex are activated during action execution and deactivated during action observation (Christian and Valeria, 2010), leading to different firing patterns when executing own actions or only observing others' actions. The existence of anti-mirror neurons can distinguish who is the producer of the emotional overt action, which is also the embodiment of preliminary self-awareness. Anti-mirror neurons were found in the M1 region in Motor Cortex, the emergence of which is due to the gating mechanism of SMA neurons (Gazzola and Keysers, 2008). SMA neurons directly project to M1 neurons; the firing state of M1 neurons is totally decided by SMA neurons. SMA neurons will produce different firing patterns during action observation and action execution, which can control M1 neurons to activate during action execution and deactivate during action observation. This process is considered to be the basis for self-other differentiation (Mukamel et al., 2010).

### 2.2. The Architecture of the Computational Model

**Figure 2:**
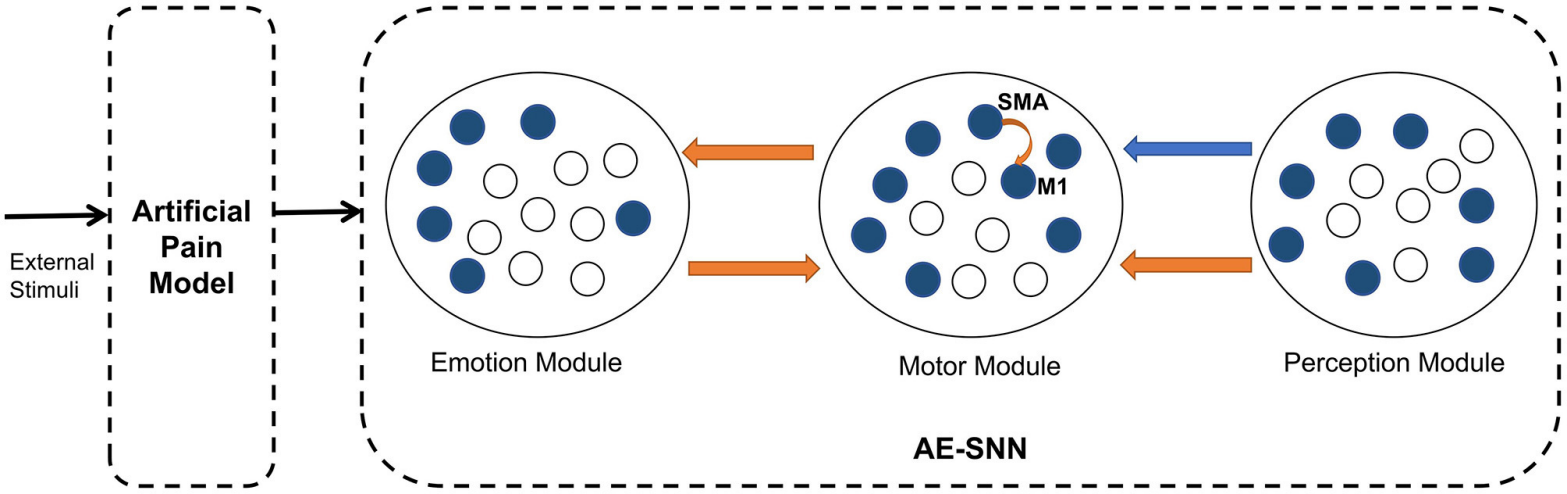
The architecture of the brain-inspired affective empathy computational model. The two dotted boxes represent two submodels: The Artificial Pain Model and the affective empathy spiking neural network (AE-SNN). Each circle represents a neuron population. The orange arrows indicate excitatory connections, and the blue arrow indicates inhibitory connections.

This subsection describes the overall architecture of the brain-inspired affective empathy computational model. As shown in Figure 2, the model is divided into two submodels: the Artificial Pain Model and the affective empathy spiking neural network (AE-SNN).

The Artificial Pain Model defines Artificial Pain from the Free Energy perspective, and it can receive different external stimuli and produce the corresponding pain state, the output of this model will be used as the input of the AE-SNN.

The AE-SNN simulates the function and connection of the brain regions mentioned in Section 2.1, which contains the Emotion Module, Motor Module, and Perception Module. Each module is a neuron population. The different emotional states are represented by population coding in the Emotion Module (Fang et al., 2021). The Motor Module represents the different emotional overt actions. The mirror neurons and anti-mirror neurons will emerge in Motor Module. Because anti-mirror neurons are formed due to the SMA neurons and M1 neurons, we set a certain number of neurons in this population to simulate the properties of M1 and SMA neurons. M1 neurons are only activated by SMA neurons, as shown by the orange curved arrow in Figure 2. The Perception Module represents the perception information of the different emotional overt actions, which are also encoded by population coding. The Motor Module and the Emotion Module are connected in a bidirectional way, all of which are excitatory connections. The connection from the Perception Module to the Motor Module contains inhibitory connections to SMA neurons and excitatory connections to other neurons. In Figure 2, the orange arrows indicate excitatory connections, and the blue arrow indicates inhibitory connections.

### 2.3. Model Implementation

#### 2.3.1. Artificial Pain as an Internal State

We propose that the traditional approaches followed by previous studies in the Affective Computing field are far from enough to achieve real artificial affective empathy. Emotion should be modeled as the bottom-up internal state of the agent so that it can process environmental information more adaptively and carry out affective empathy more reasonably. This subsection discusses the modeling of a particular emotion–pain.

As mentioned in Subsection 2.1, the first step of the Artificial Pain Model is to quantify the actual damage of intelligent agents. From the perspective of Free Energy, body damage is an unexpected state, called the entropy increasing state (Friston, 2010). However, the entropy of organisms cannot be directly quantified; the Free Energy is the upper bound of entropy, and minimizing Free Energy approximates minimizing the entropy (Ramstead et al., 2018). Thus, FEP is a way to quantify the damage.

Bogacz demonstrated the relationship between Free Energy and entropy (Bogacz, 2017), providing the definition and expression of Free Energy in detail: the brain could not directly know the actual state of the external world ϕ; it can only constantly estimate the state of the external world $\hat{\phi }$; the sensory information *s* received by the sense organs is used to verify the estimation. The Free Energy is finally simplified and expressed as the negative logarithm of the joint probability distribution of the state estimation $\hat{\phi }$ and the sensory information *s*. According to the definition, the larger the joint probability distribution, the smaller the Free Energy, and the more accurate the estimation. Expanded by the Bayesian formula $p\left(s,\hat{\phi }\right)=p\left(s|\hat{\phi }\right)p\left(\hat{\phi }\right)$:


(1) $$\begin{array}{l} FE=-\ln p(s,\hat{\phi })\\ \;=-\ln p(s|\hat{\phi })-\ln p(\hat{\phi }) \end{array}$$

We replace the probability distribution with a Gaussian distribution *f*(*s*; *g*(.), σ) with mean *g*(.) and variance σ, supposing σ = 1:


(2) $$\begin{array}{l} EF=-\ln\;f_{1}(s;g_{s}(\hat{\phi }),\sigma_{s})-\ln\;f_{2}(\hat{\phi };\mu_{\phi },\sigma_{\phi })\\ \;=\frac{{\left[s-g_{s}(\hat{\phi })\right]}^{2}}{2\sigma_{s}}+\frac{{\left[\hat{\phi }-\mu_{\phi }\right]}^{2}}{2\sigma_{\phi }}-\frac{1}{2}\ln\;\sigma_{s}-\frac{1}{2}\ln\;\sigma_{\phi }\\ \;=\frac{1}{2}{\left[s-g_{s}{\left(\hat{\phi }\right)}^{2}+\frac{1}{2}\hat{\phi }-\mu_{\phi }\right]}^{2} \end{array}$$

The first item in Equation 2 is the sensory dynamic (McGregor et al., 2015). *g*_*s*_() is the sensory generating function, mapping the relationship between the world state and the sensory information learned by experience in advance. The brain actively estimates the world state $\hat{\phi }$, then predicts sensory information through the generating function $g_{s}\left(\hat{\phi }\right)$. The actual sensory information *s* received through the sense organs is used to calculate prediction errors. The second term in Equation 2 is the environmental dynamic (McGregor et al., 2015). The estimation of the brain depends on prior knowledge μ_ϕ_ of the environment and this item is similar to the prior error of Bayes theorem. Environmental dynamic and sensory dynamic jointly determine the value of Free Energy.

For robotic systems, the world state mentioned above is the body state of the robot and the sensory information for robots are vision *s*_*v*_ and proprioception *s*_*p*_ (Lanillos and Cheng, 2018), as shown in Equation 3. The robot continuously estimates its body state $\hat{\phi }$, and predicts the vision $g_{sv}\left(\hat{\phi }\right)$ and proprioception $g_{sp}\left(\hat{\phi }\right)$, respectively. The generating functions *g*_*sv*_() and *g*_*sp*_() can be learned by motor babbling in advance (Lanillos and Cheng, 2018), which is the mapping relationship between the body state and the vision information or proprioception information. Therefore, the sensory dynamic of the robotic system includes visual prediction error and proprioception prediction error. For the environmental dynamic, the body state ϕ of the robot at the current moment is determined by the state ϕ and the action *a*′ at the last moment. *g*_ϕ_() represents the mapping relationship. When the robot's body structure is deformed, or the sensor is damaged, the sensory dynamic will be affected. When the robot motor structure is damaged, the environmental dynamic will be affected. In these cases, the value of Free Energy will change. So the value of Free Energy is taken to evaluate the robot's body damage.


(3) $$\begin{array}{l} FE=\frac{1}{2}{\left[s_{v}-g_{sv}\left(\hat{\phi }\right)\right]}^{2}+\frac{1}{2}{\left[s_{p}-g_{sp}\left(\hat{\phi }\right)\right]}^{2}+\frac{1}{2}{\left[\hat{\phi }-g_{\phi }\left(\phi^{\prime },a^{\prime }\right)\right]}^{2} \end{array}$$

In living organisms, physical damage can result in a pain state, and physical damage can be quantified by the value of Free Energy. Thus, in a robotic or virtual agent system, Artificial Pain can be considered relative to the value of Free Energy that represents their body damage. When the Free Energy is greater than 0, the body is in an injured state and then causes a pain state; When the Free Energy equals 0, the body is in a normal state.

In some potential dangerous scenarios, substantial injury has not occurred, but the organisms can also feel pain. This is because the brain can associate the experience of pain with injury-related cues so that it can quickly avoid similar cues. In the actual application of the robot, after the robot suffered substantial body damage, the robot's vision system should capture the corresponding dangerous scenarios. The robot will then associate it with the internal pain state and avoid potential damage. The significance of pain is that it is a warning signal for the actual and potential body damage of the living organisms to protect themselves (Melzack, 2001). The Artificial Pain created with the proposed model can not only generate a pain warning signal when the body damage actually occurs, but also avoid potential damage in the future, achieving the same significance as the living organisms' pain.

#### 2.3.2. The Affective Empathy Spiking Neural Network

When different internal emotional states are generated through the Artificial Pain Model, the AE-SNN simulating mirror mechanism will be trained. This subsection describes the concrete design and implementation of the AE-SNN.

**1. LIF neuron model and STDP learning rule**. Compared to conventional artificial neural networks, spiking neural network (SNN) captures more essential characteristics of the biological brain (Ghosh-Dastidar and Adeli, 2009; Khalil et al., 2017, 2018a; Zhao et al., 2018). In recent years, SNN has been successfully applied in many aspects of cognitive function modeling, such as decision making and creative processes (Héricé et al., 2016; Khalil and Moustafa, 2022). The Leaky Integrate-and-fire (LIF) neuron is a well accepted computational model for SNN neurons (Tal and Schwartz, 1997), and we use it as the basic unit of our model. The LIF neuron dynamics are described by the following Equation 4 and Equation 5:


(4) $$\begin{array}{l} \tau_{m}\frac{du}{dt}=-\left[u_{t}-u_{rest}\right]+RI\left(t\right) \end{array}$$

(5) $$\begin{array}{l} \underset{\delta \to 0}{\lim}u\left(t^{f}+\delta \right)=u_{reset} \end{array}$$

*u*_*t*_ is the membrane potential of the neuron at time *t*, *u*_*rest*_ is the membrane potential at steady-state, *R* is the resistance, *I*(*t*) is the input current, and τ_*m*_ is the time constant. When the membrane potential *u*_*t*_ exceeds a certain threshold *u*_*th*_, the neuron fires and *t*_*f*_ is the firing time. Once the neuron has fired, the membrane potential returns to its reset state *u*_*reset*_ (Fang et al., 2021). In this article, the parameters of the LIF neuron are: *u*_*rest*_ = *u*_*reset*_ = 0*mV*, τ_*m*_ = 30*ms*, *u*_*th*_ = 60*mV*.

We use Spike Timing Dependent Plasticity (STDP) as a synapse learning rule to update synaptic weights. STDP is the most basic learning method in the brain, which relies on the time difference between the firing of pre-synaptic neurons and post-synaptic neurons to train the synaptic weight (Caporale and Dan, 2008). The weight update can be written as Equation 6:


(6) $$\Delta \omega =\left\{ \begin{array}{l} A^{+}exp(\frac{\Delta t}{\tau^{+}}),\;\Delta t<0\\ -A^{-}exp(\frac{-\Delta t}{\tau^{-}}),\;\Delta t>0 \end{array}\right.$$

Here, Δ*t* = *t*_*i*_−*t*_*j*_, *t*_*i*_ and *t*_*j*_ are the time of the firing of the pre-synaptic neuron and the post-synaptic neuron, respectively (Zhao et al., 2018). *A*^+^ and *A*^−^ are the learning rates. τ_+_ and τ_−_ are the time constants. According to this rule, the connections will be strengthened when pre-synaptic neurons fire before post-synaptic neurons and will be weakened when pre-synaptic neurons fire after post-synaptic neurons. Here, τ_+_ = τ_−_ = 10*ms*, *A*^+^ = 0.25, *A*^−^ = 0.01.

**2. The training process**. We describe the training process of AE-SNN from two aspects: First, set the value of fixed weights in the three modules. In the Motor Module, the excitatory synaptic weights between SMA neurons and M1 neurons are set so that once the SMA neurons are activated, the connected M1 neurons are activated immediately. In addition, The Emotion Module connects all neurons in the Motor Module except M1 neurons. We set these fixed synapse weights because the different emotion overt actions are determined by hereditary genetic factors (Darwin, 2015). Different emotion neurons' firing will lead to different motor neurons' firing. Since the connection between the two modules is bidirectional, these synapse weights can also be used in reverse when known the activation of the Motor Module to infer the activation of the Emotion Module.

**Figure 3:**
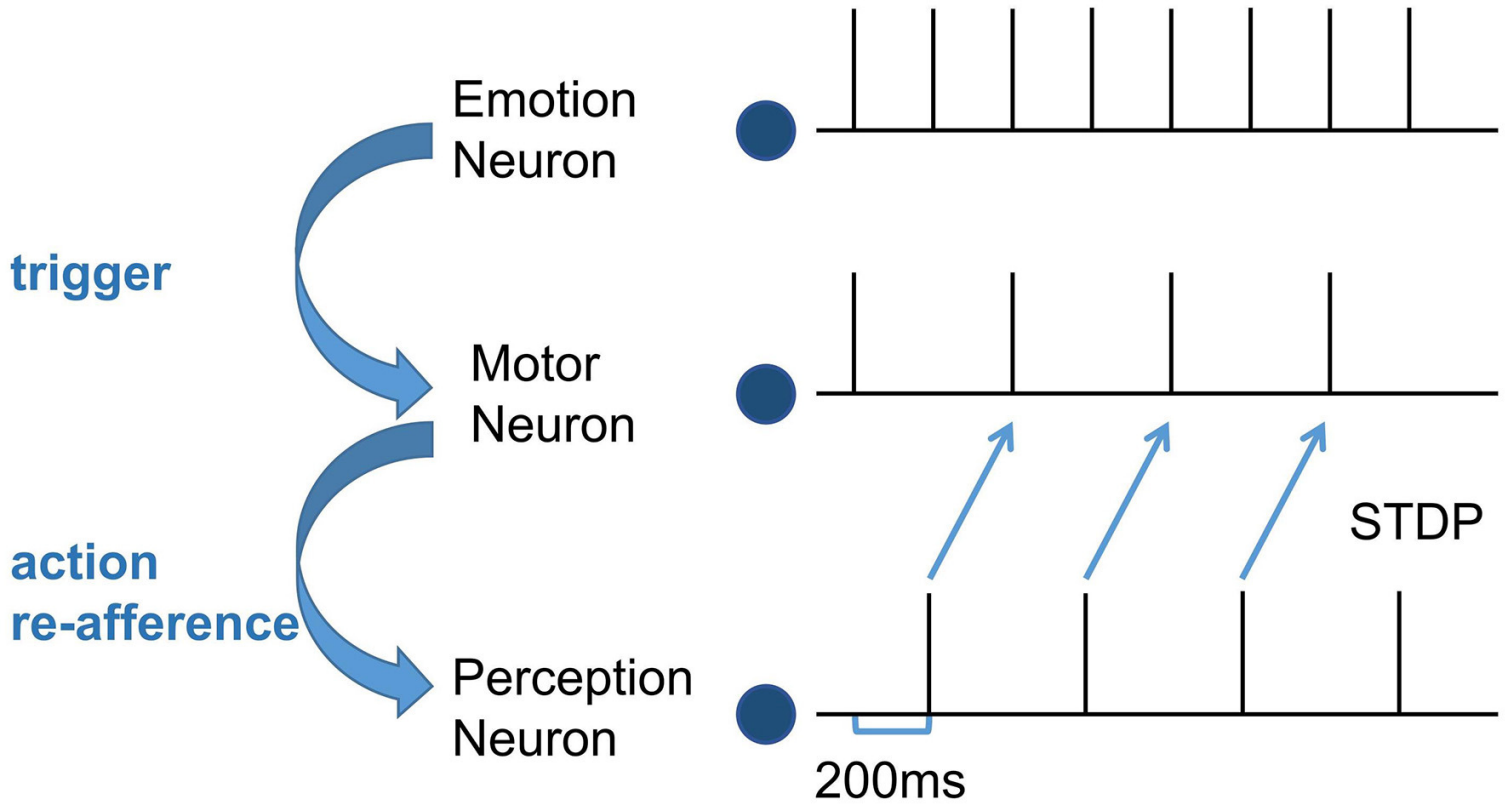
The spike sequence of the three module neurons and the STDP training process between the Motor neuron and the Perception neuron. The firing of the Emotion neuron triggers the firing of the corresponding Motor neuron. As a result of action re-afference, the Perception neuron subsequently fires. There is a 200 ms delay from the firing of the Motor neuron to the firing of the Perception neuron. The blue curved arrows indicate the causality of the three modules, and the blue straight arrows represent the STDP training process.

Second, train the synapse weights between the Perception Module and the Motor Module. For the emergence of mirror neurons, we simulated the process of action execution and action re-afference and used the STDP learning rule to adjust the synapse weights. It takes 100 ms from the firing of the motor neurons to action execution, 100ms from the hearing/watching of that action to trigger the firing of the perception neurons, and the total time delay is 200 ms (Keysers and Gazzola, 2014). In the training process, the emotion neurons fire at first, the corresponding motor neurons will fire and execute the emotional overt action, and after 200 ms, the corresponding perception neurons will fire. Because action execution is a continuous process, the firing of the motor neurons is still going on, as shown in Figure 3. Due to this temporal correlation, the excitatory connections between the motor neurons executing the action and the perception neurons representing the same action will be strengthened by STDP, forming the mirror neurons in the Motor Module. In the emergence process of anti-mirror neurons, due to the connections between the SMA neurons and the perception neurons being inhibitory, the inhibitory connections between the SMA neurons and the perception neurons representing the same action will be strengthened by STDP. However, M1 neurons are only activated by SMA neurons, so M1 neurons are deactivated after the SMA neurons are inhibited, and the connection between the M1 neurons and the perception neurons will not be established. The M1 neurons are only activated during action execution, reproducing the property of anti-mirror neurons.

In the training stage, the connections among the three modules are established, and the mirror neurons and anti-mirror neurons emerge. When perceiving the emotional overt action of others, the perception information of overt action will be encoded in the Perception Module as input. Then it will activate the mirror neurons in the Motor Module, and further activate the corresponding emotion neurons to empathize with others.

### 2.4. Intrinsic Motivation for Altruistic Behavior

**Figure 4:**
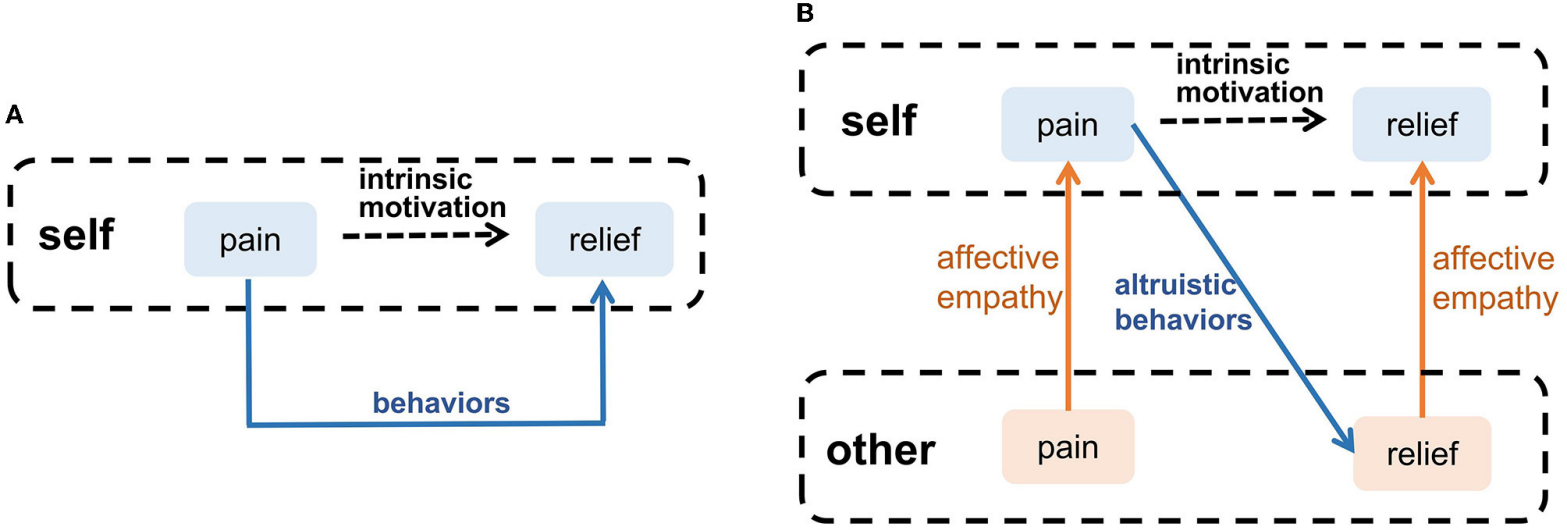
The intrinsic motivation of altruistic behavior. **(A)** Self-pain relief. **(B)** Altruistic behaviors that relieve others' pain.

Waal and Preston proposed that altruistic behavior derives from intrinsic motivation, and affective empathy is a major factor to generate this motivation (Waal and Preston, 2017). Many psychologists argued that emotion is the motivation for most decisions, guiding individuals to avoid negative emotions (such as pain and sadness), which is an intrinsic motivation for survival, such as eating (Lerner et al., 2015). When people have negative emotions, they will adopt adaptive behaviors to eliminate negative emotions, as the blue arrow shown in Figure 4A. When observing others' negative emotions, the ability of affective empathy can transfer the others' negative emotions to the observer, as the orange arrows shown in Figure 4B. This shared negative emotion provides intrinsic motivation for the observer's altruistic behavior, In order to eliminate shared negative emotion, the observer will try to adopt altruistic behavior actively, such as help, and consolation, as the blue arrow shown in Figure 4B. When the others' negative emotion is eliminated, the observer's shared negative emotion is indirectly eliminated by affective empathy. Therefore, Altruistic behavior originates from spontaneous intrinsic motivation. Affective empathy can provide this motivation by realizing emotion transfer.

After exploring the intrinsic motivation of the altruistic behavior, we combine the brain-inspired affective empathy computational model proposed above to complete an altruistic rescue task and achieve the rescue of companions by an intelligent agent in the grid world. In our model, the AE-SNN has self-other differentiation ability. When the intelligent agent has negative emotion, if the negative emotion comes from itself, it will act on itself; if the negative emotion comes from others, it will act to help the others and, thus, indirectly help itself.

## 3. Experiment

This subsection introduces the applications of the brain-inspired affective empathy computational model on intelligent agents in the grid world environment. Bartal et al. studied that rats learned to open doors to rescue their companions trapped in containers through trial and error (Bartal et al., 2011). We designed the experiment inspired by this behavior paradigm. The experiment process is divided into two phases: (1) The development of pain empathy ability. We designed an agentA to explore the grid world and experience pain state through the Artificial Pain Model. Then train its AE-SNN to develop its pain empathy ability. (2) The altruistic rescue task. We design a two-agent task, in which agentA uses the trained AE-SNN to empathize with agentB and implements rescue behavior driven by intrinsic altruistic motivation.

### 3.1. Environment Settings

**Figure 5:**
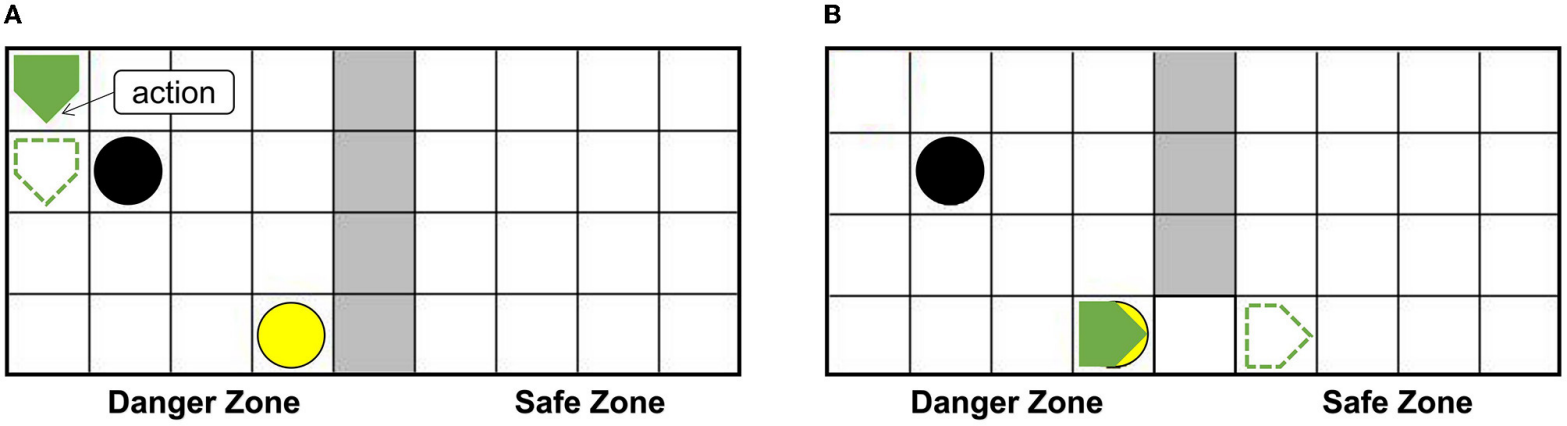
The setups of the grid world. The green pentagon represents the agent. The direction pointed by the tip of the pentagon is the direction of the agent's next action. The gray area is walls, the danger zone is on the **(A)**, and the safety zone is on the **(B)**. The yellow circle is the switch. The black circle in the danger zone is a dangerous object.

The setups of the grid world are shown in Figure 5A. The green pentagon represents the agent. The direction pointed by the tip of the pentagon is the direction of the agent's next action, and the green dotted line represents the next position. The gray area in the middle is walls, splitting the danger zone on the left and the safety zone on the right. The black circle in the danger zone is a dangerous object, which can lead to damage when the agent crashes into it. The yellow circle is the switch. When the switch is turned on, a passage from the danger zone to the safety zone can be established as shown in Figure 5B, then the agent can enter the safety zone spontaneously and immediately, and the damaged agent will recover to the normal state (just like when the container is opened the trapped rat will quickly leave the container, as mentioned in the Bartal et al., 2011). In the action execution process, the agent can detect its internal state through the Artificial Pain Model. For the AE-SNN of the agent, the number of neurons in the Emotion Module, Motor Module, and Perception Module are 40, 50, and 40, respectively. When different internal states arise, the corresponding neurons of the Emotion Module of AE-SNN will fire, activating the neurons of the Motor Module to produce corresponding emotional overt actions. In our experiments, we designed the color change of the agent to be equivalent to emotional overt actions (e.g., human facial expressions or crying). The agent shows red in a pain state and green in a normal state. Agents can use their own Perception Module to perceive their own and others' emotional overt actions, just as people can hear their own and others' cries. The trained AE-SNN makes the agent have empathy ability.

### 3.2. The Developing of Pain Empathy Ability

#### 3.2.1. Experimental Procedure

We place dangerous objects, a switch, walls, and an agentA in the grid world. The agentA explores the environment and uses the Artificial Pain Model to detect its own internal state, completing the training of the AE-SNN.

We set the damage rule of agent: when an agent collides with a dangerous object, its ability to act will be impaired. For example, if a command is given to the right, the agent actually moves a unit to the left. For the Artificial Pain Model, the 'environmental dynamic' of the Free Energy will result in errors, as the third item in Equation 3. We first set the mapping relationship $g_{\phi }\left(\phi^{\prime },a^{\prime }\right)$ of the Artificial Pain Model, which is the moving rule of the agent: When the agent performs an action, its horizontal and vertical coordinates will change accordingly. For example, if an upward action is performed, its vertical-coordinate's value reduces, and if a rightward action is performed, its horizontal-coordinate's value adds. The agent knows its moving rule in advance and uses this rule to continuously and actively predict its next position. When the prediction is wrong, the Free Energy is greater than 0, indicating that damage occurs and the agent will be in the pain state.

**Figure 6:**
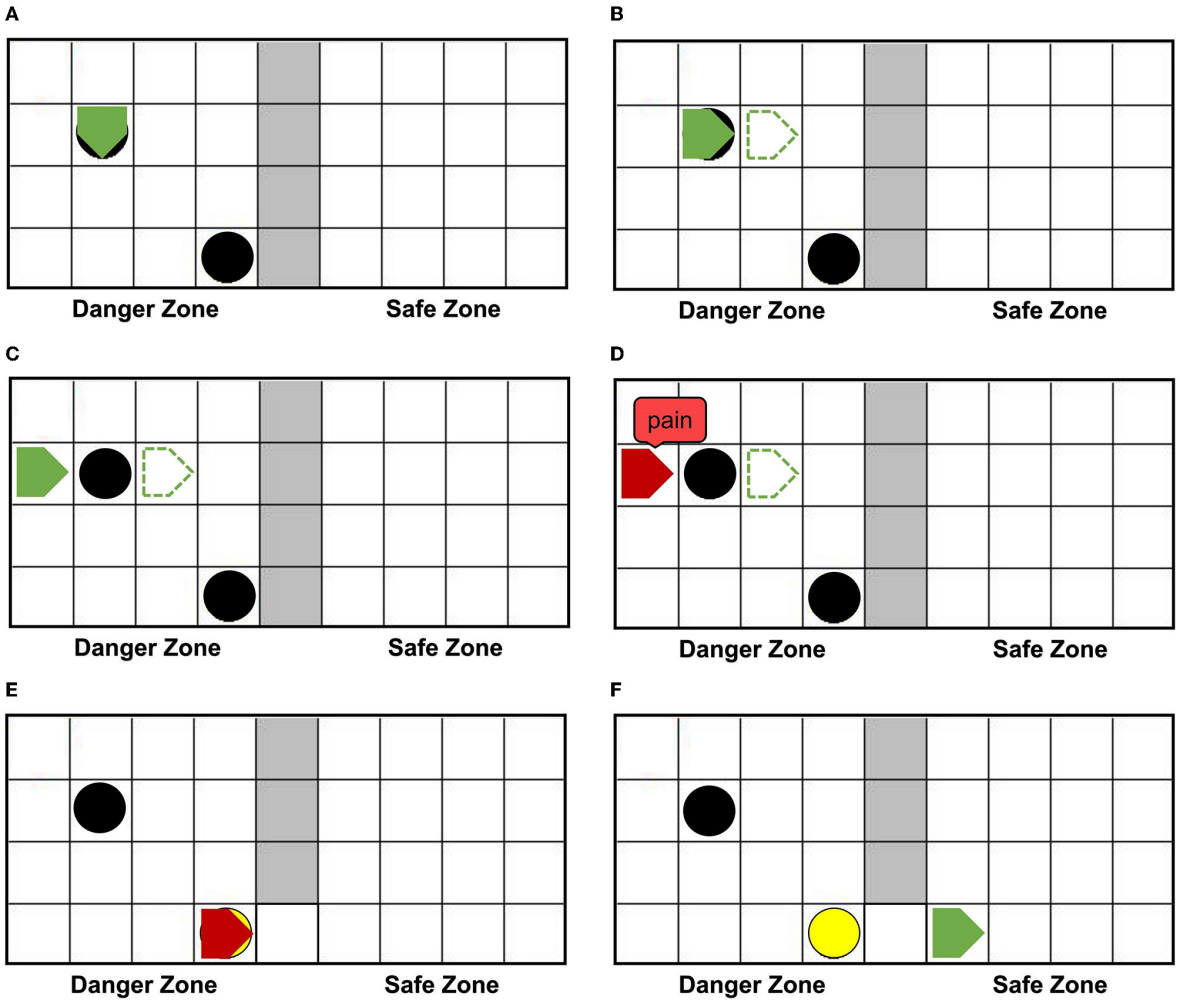
The random exploration process of agentA. The agentA explores the environment and uses the Artificial Pain Model to detect its own internal state, completing the training of the AE-SNN. **(A,B)** Show that agentA collides with the dangerous object during the exploration. **(C,D)** Show that agentA generates a motor damage and is detected by the Artificial Pain Model; agentA is in the pain state and turnsred. **(E,F)** Show that the switch being touched, establishing a pathway from the danger zone to the safety zone; agentA then enters the safety zone and returns to the normal state.

Figure 6A shows that agentA is colliding with the dangerous object. Figure 6B shows that the next motion command of agentA is toward the right, the prediction is the position of the green dotted line. But Figure 6C shows that the actual action of the agentA is toward the left. At the same time, the motor damage will be detected by the Artificial Pain Model, and the agentA will be in the pain state. After the pain state generates, the first 20 neurons in the Emotion Module that represent pain will fire and activate the neurons in the Motor Module to produce painful overt action. In this experiment, the color of the agentA turns red, as shown in Figure 6D. After that, the agentA's Perception Module will perceive its own color information. The first 20 neurons of the Perception Module that represent perceiving red will be activated, completing a training epoch of AE-SNN in the pain state by STDP. After 100 training epochs, the AE-SNN finishes training, and the agent has pain empathy ability. Figures 6E,F show that agentA touches the switch during exploration and establishes the passage from the danger zone to the safety zone. Then it enters the safety zone and recovers to its normal state. These two figures aim to illustrate the role of the switch.

#### 3.2.2. Results Analysis

**Figure 7:**
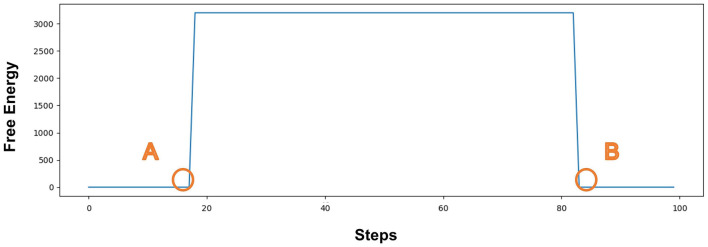
The value of Free Energy. The X-axis represents the number of steps and Y-axis represents the value of Free Energy. Point (A) indicates that the agentA collides with a dangerous object at 19 steps, and the damage generates; Point (B) indicates that the agent enters the safe zone at 82 steps and recovers to its normal state.

Figure 7 represents the value of Free Energy during exploration. The X-axis represents the number of steps. The Y-axis represents the value of Free Energy. The value of Free Energy is the sum of the square of the difference between the predicted agentA's coordinates and the actual agentA's coordinates. We designed the agent as a pentagon, each vertex has a 2D coordinate (*X, Y*), so the agent has five sets of 2D coordinates at each position, i.e., (*X*_1_, *Y*_1_), (*X*_2_, *Y*_2_)...(*X*_5_, *Y*_5_). The Free Energy is equal to the sum of the squares of the differences in the corresponding coordinates, as described in Equation 7. The unit grid length is 25, resulting in the Free Energy range of [3125,12500]. Since our model is only related to whether the Free Energy is greater than 0, we put all values that are greater than 3125 equal to 3125. PointA indicates that the agentA collides with a dangerous object at 19 steps, and the damage generates. The Free Energy is greater than 0, causing the agentA to be in the pain state. PointB indicates that the agentA enters the safe zone at 82 steps, and recovers its normal state. The Free Energy is equal to 0, causing the agentA's pain state to be alleviated.


(7) $$\begin{array}{l} FE=\overset{5}{\underset{x=1}{\sum }}{\left(X_{pre}-X_{actual}\right)}^{2}+\overset{5}{\underset{y=1}{\sum }}{\left(Y_{pre}-Y_{actual}\right)}^{2} \end{array}$$

**Figure 8:**
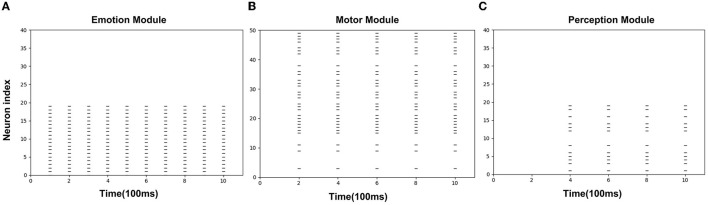
The spikes of the neurons in the Emotion Module **(A)**, Motor Module **(B)**, and Perception Module **(C)** in the training phase of AE-SNN. The X-axis represents the firing time, and each unit represents 100 ms. Y-axis represents the index of the neurons.

Figures 8A–C is the spike diagrams of the three modules of AE-SNN in the pain state. The X-axis represents the firing time, and each unit represents 100 ms. The Y-axis represents the index of the neurons. Figure 8A represents the firing of neurons that represent the pain state in the Emotion Module. Figure 8B shows the firing of neurons that represent the painful overt action in the Motor Module as the agentA's color turns red. Figure 8C shows the firing of neurons in the Perception Module, which corresponds to agentA perceiving its color information. The firing of the Emotion Module will then trigger the firing of the Motor Module by setting weights in advance, and then the Perception Module will fire after a 200ms delay, indicating the time interval for action execution and action re-afference. With the time correlation, the connections between the Perception Module and the Motor Module can be established by STDP.

**Figure 9:**
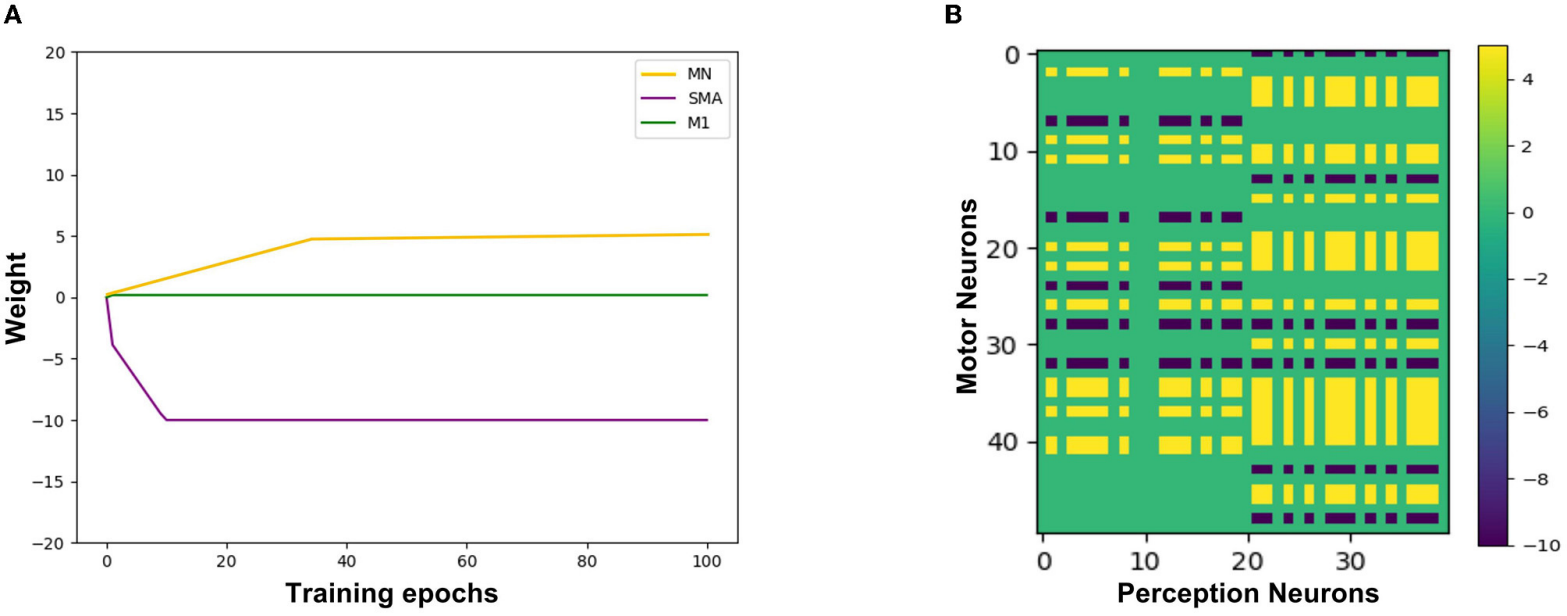
The synaptic weights from the Perception neurons to the Motor neurons. **(A)** The change of synaptic weights. The X-axis represents the training epochs and Y-axis represents the value of weights. **(B)** The trained value of synaptic weights. The X-axis represents the index of Perception neurons and Y-axis represents the index of Motor neurons.

Figure 9A shows the synaptic weights training process from the Perception Module to the Motor Module. The X-axis represents the training epochs. The Y-axis represents the value of weights. The purple line indicates the change of inhibitory weights between the Perception neurons and the SMA neurons. The green line indicates a change of weights between the Perception neurons and the M1 neurons, and due to the gate mechanism of the SMA neurons, the weight is maintained near 0, which is equivalent to not establishing synaptic connections. The yellow line indicates the change of excitatory weights between the Perception neurons and other neurons in the Motor Module, and the value of the weight gradually increases, indicating that these neurons are mirror neurons. Figure 9B shows the trained synaptic weights between the Perception Module and the Motor Module. The X-axis represents the index of Perception neurons. The Y-axis represents the index of Motor neurons. The color represents the value of the weight. The purple areas represent inhibitory synaptic weights between the Perception neurons and the SMA neurons. The yellow areas represent excitatory synaptic weights between the Perception neurons and the mirror neurons that represent the same emotional overt action. The green areas represent unestablished synaptic connections, which contain the connections between M1 neurons and Perception neurons.

### 3.3. The Altruistic Rescue Task

#### 3.3.1. Experimental Procedure

**Figure 10:**
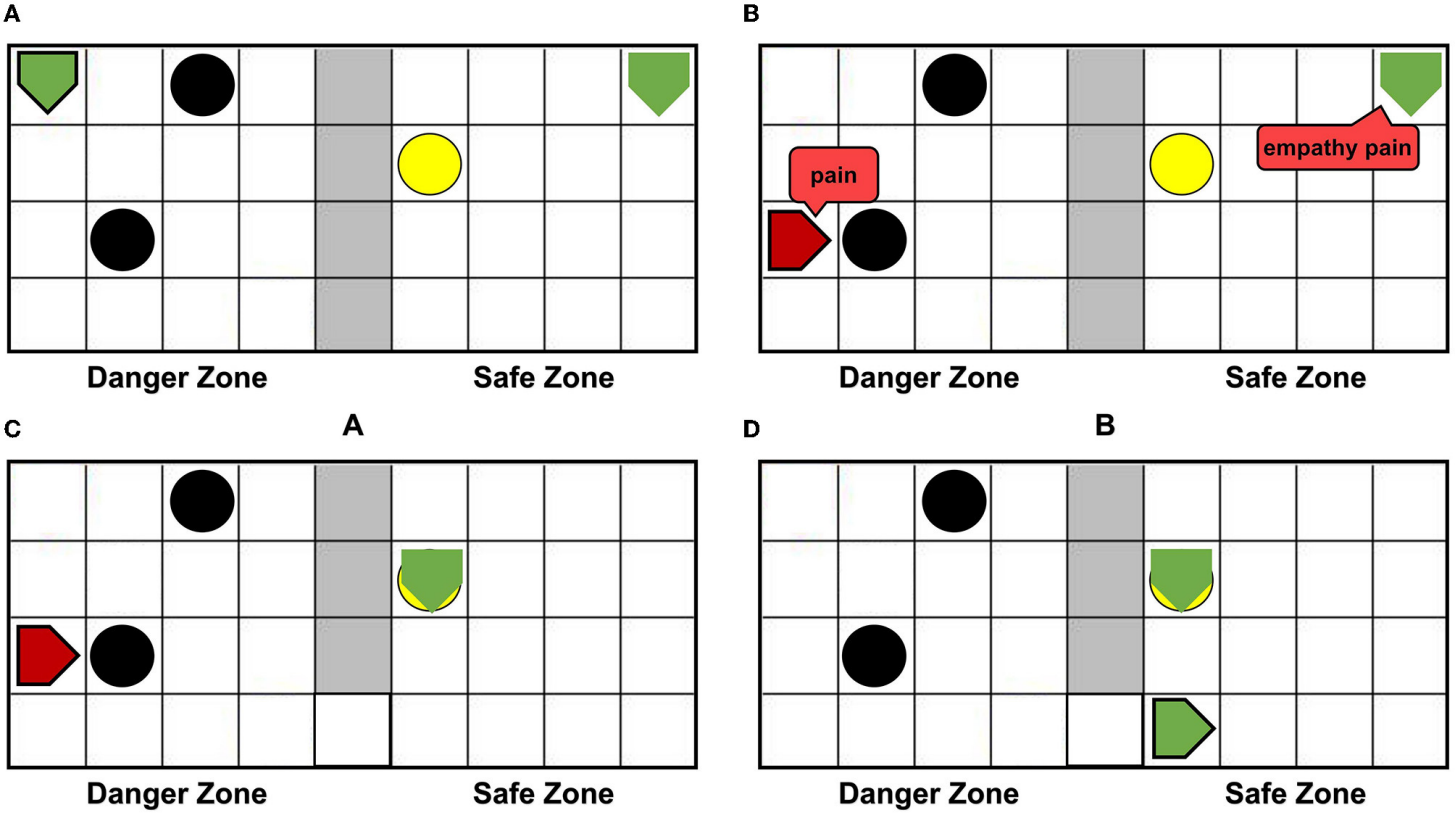
The altruistic rescue process. The agentA through AE-SNN empathizes with agentB and carries out rescue behavior. **(A)** Shows that agentA with affective empathy ability is placed in the safe zone and agentB (green pentagon with black border) is placed in the danger zone for exploration. **(B)** Shows that agentB inevitably collides with a dangerous object, causing the pain state and turning red; agentA generates pain empathy through AE-SNN. **(C)** Shows that agentA learns to find the switch. **(D)** Shows that the switch is touched, agentB enters the safe zone and relieves the pain state.

Using the AE-SNN trained in Section 3.2, we designed an altruistic rescue task. Figure 10A shows that two agents, a dangerous object, a switch, and walls are placed in the grid world. In the previous task, the agentA with affective empathy ability is placed in the right safety zone, and agentB (green pentagon with black border) is placed in the danger zone for exploration. We place the yellow switch on the side of the safety zone, so agentB has no self-rescue ability and could only be rescued through agentA.

When agentB explores the environment, it will inevitably encounter a dangerous object, resulting in pain and turning into red color, as shown in Figure 10B. AgentA will perceive the color information of agentB, then generate empathy pain through AE-SNN. The intrinsic altruistic motivation will drive agentA to take action to rescue agentB and find the optimal strategy by the reinforcement learning method. Eventually, agentA learned to go to the switch as soon as possible and establish the passage from the danger zone to the safety zone, as shown in Figure 10C. After the switch is turned on, agentB will enter the safety zone and relief the pain state, then agentA's empathy pain will also be relieved through AE-SNN as shown in Figure 10D, completing the rescue of the companion.

#### 3.3.2. Results Analysis

**Figure 11:**
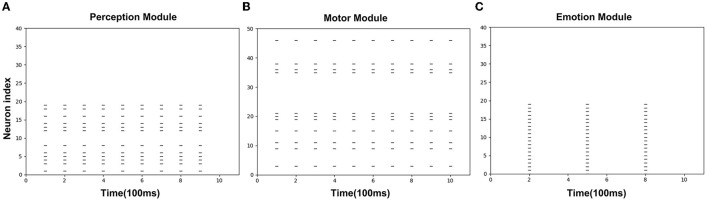
The spikes of the neurons in the Perception Module **(A)**, Motor Module **(B)**, and Emotion Module **(C)** in the altruistic rescue task phase. The X-axis represents the firing time and each unit represents 100 ms. Y-axis represents the index of the neurons.

Figures 11A–C shows the spike diagrams of the three modules of the agentA during the altruistic rescue process. Figure 11A shows the firing of the neurons in the Perception Module, representing agentB's color information. Figure 11B shows the firing of the mirror neurons in the Motor Module, which realizes the understanding of the pain state at the motor level. Figure 11C shows the firing of the neurons in the Emotion Module, which realizes the understanding of the pain state at the emotional level. Moreover, in this process, compared with Figure 8B mentioned in Section 3.2.2, not all neurons in the motor area are activated (in fact only mirror neurons are activated), so the conditions for executing painful overt actions do not arrive. Therefore, although the agentA feels pain, it does not turn red.

## 4. Discussion

This article focuses on the brain-inspired affective empathy computational model and its application. Inspired by the neural mechanism of affective empathy, we simulated the generation process of pain, trained the emergence of mirror neurons and anti-mirror neurons, and completed the altruistic rescue task. In this section, we will discuss the strengths and limitations of our computational model and its application prospects.

In the AE-SNN, we reproduced the emergence process of mirror neurons and anti-mirror neurons. Inspired by the gating mechanism from SMA to M1 proposed in the neuroscience literature (Christian and Valeria, 2010), we also set up neurons with similar functions in the Motor Module of AE-SNN. This eventually led to eight main types of neurons in the Motor Module, as shown in Table 1. If a neuron is activated when executing action1 and observing action1, it is the mirror neuron of action1, which is used to understand emotional overt action1. If a neuron is activated when executing action2 and observing action2, it is the mirror neuron of action2, which is used to understand emotional overt action2. If a neuron is activated during the execution of both action1 and action2 and is deactivated during the observation of both action1 and action2, it is the anti-mirror neuron, which is used to distinguish between self and others and avoid unnecessary emotional overt actions in the affective empathy process.

**Table 1:** The analysis of the eight main neuron types in the Motor Module.

	**Action1**	**Action2**	**Perception1**	**Perception2**	**Type**
1	√				
2	√		√		MN(1)
3		√			
4		√		√	MN(2)
5	√	√	√		MN(1)
6	√	√		√	MN(2)
7	√	√	√	√	MN(1,2)
8	√	√			ANTI-MN

*The MN refers to mirror neuron and the ANTI-MN refers to anti-mirror neuron. √ indicates whether the neuron is activated under these four conditions (Action 1, Action 2, Perception 1, Perception 2)*.

Gazzola and Keysers studied the fMRI data of human subjects and concluded that mirror neurons were reliably observed in the premotor cortex (PM), the supplementary motor cortex (SMA), and other brain regions, and anti-mirror neurons were observed in the M1. They also discussed the gating mechanism of the SMA neurons (Gazzola and Keysers, 2008). Mukamel et al. also illustrated a similar conclusion, and further discussed that anti-mirror neurons can maintain self-other differentiation (Mukamel et al., 2010). The results in Table 1 show that the AE-SNN produces neuron types consistent with these two references.

In the altruistic rescue task, agentA's rescue path optimization used reinforcement learning. Unlike traditional reinforcement learning, the reward function in this task is not an extrinsic reward set by the human in advance, but an intrinsic reward obtained through the agent's internal model. First, agentB generates pain states through the Artificial Pain Model, and agentA generates empathy pain through its AE-SNN. When agentA touches the yellow switch accidentally, the passage from the danger zone to the safety zone will be established, then agentB will enter the safe zone, the pain will be relieved, and then the pain empathized by agentA will be relieved. For the brain, the relief of negative emotions can be seen as an intrinsic reward (Porreca and Navratilova, 2017). Thus, agentA marks the yellow switch as a positive reward. Then it can continuously optimize its Q-table to learn the optimal rescue path based on this reward.

There are some limitations to our study. First, there is a simplification in the design of anti-mirror neurons. In fact, the gate mechanism of SMA neurons is complex (Gazzola and Keysers, 2008), which is not easy to realize for computational modeling. Our study could be seen as the first step toward a more biologically realistic computational model of anti-mirror neurons from a functional perspective. In addition, there is some simplification in the process of artificial emotion generation. Emotion is a very complex cognitive function of organisms involving the regulation of a variety of hormones and neural circuits (Lövheim, 2012). The Artificial Pain Model proposed in this article is just a prototype. In the future, we will study more about the nature of emotion and the relationship between emotion and emotional overt actions (Mirabella, 2018; Mancini et al., 2020, 2022; Mirabella et al., 2022).

Currently, our study is only to realize the application of the mirror mechanism in a virtual environment. In the social robots field, the mirror mechanism is also necessary (Asada, 2015). If the robot associates its emotion with the perception information of the corresponding emotion overt action, it will understand its companion's emotion when perceiving similar emotion overt information from the companion. This mirror mechanism can help robots to empathize with their companions. We will explore the application of these mechanisms in robots in the future.

The prediction process in the Artificial Pain Model simulates the evolution of pain. Organisms evolved pain by experiencing physical damage (Walters and Williams, 2019). We use the Free Energy Principle (FEP) to model this detection process of physical damage: The brain detects abnormal sensations through continuous prediction of multiple physical sensations. That is a prediction at the physical sensation level. It is worth noting that the brain also has predictions at the event level, such as predicting that the organism will be injured. This is essentially a prediction of whether an injury event will occur, which is different from the prediction at the physical sensation level in the Artificial Pain Model.

In summary, we proposed a brain-inspired affective empathy computational model, which involves the generation process of Artificial Pain and the reproduction of the mirror mechanism of MNS, along with the self-other differentiation ability. We apply this model to achieve pain empathy and altruistic behavior in the virtual grid world environment. We hope that our study will contribute to the harmonious coexistence among robot groups and between humans and robots.

## Data Availability Statement

The original contributions presented in the study are included in the article/supplementary material, further inquiries can be directed to the corresponding author.

## Author Contributions

HF and YZ designed the study, and performed the experiments. HF, YZ, and EL developed the algorithm and performed the result analysis, wrote and revised the manuscript. All authors contributed to the article and approved the submitted version.

## Funding

This work was supported by the New Generation of Artificial Intelligence Major Project of the Ministry of Science and Technology of the People's Republic of China (Grant No. 2020AAA0104305), the Strategic Priority Research Program of the Chinese Academy of Sciences (Grant No. XDB32070100), and the Beijing Municipal Commission of Science and Technology (Grant No. Z181100001518006).

## Conflict of Interest

The authors declare that the research was conducted in the absence of any commercial or financial relationships that could be construed as a potential conflict of interest.

## Publisher's Note

All claims expressed in this article are solely those of the authors and do not necessarily represent those of their affiliated organizations, or those of the publisher, the editors and the reviewers. Any product that may be evaluated in this article, or claim that may be made by its manufacturer, is not guaranteed or endorsed by the publisher.
